# Predictors of multidrug resistant tuberculosis among adult patients at Saint Peter Hospital Addis Ababa, Ethiopia

**DOI:** 10.11604/pamj.supp.2016.25.2.9203

**Published:** 2016-11-26

**Authors:** Muluken Dessalegn, Ermias Daniel, Sileshi Behailu, Maereg Wagnew, Josephat Nyagero

**Affiliations:** ^1^Amref Health Africa in Ethiopia, Maternal Newborn and Child Health Department, Debre Berhan, Ethiopia; ^2^Gambi University College, Addis Ababa, Ethiopia; ^3^Freelance, USA; ^4^Ministry of Health in Ethiopia, Public Health Disease prevention and control, Addis Ababa, Ethiopia; ^5^Amref Health Africa, Headquarters, Research programme, Nairobi, Kenya

**Keywords:** MDR-TB, predictors, adults, Ethiopia, HIV, tuberculosis

## Abstract

**Introduction:**

The emergence of multi-drug resistant tuberculosis (MDR-TB) has become a major public health concern that threatens advances made in global TB control efforts. Though the problem is prevalent, it did not receive major attention to generate supportive evidence for the prevention and control of MDR-TB. The aim of this study was to identify predictors of MDR-TB in a national TB referral centre in Ethiopia.

**Methods:**

An unmatched, case-control study was conducted at St. Peter Hospital to assess risk factors associated with MDR-TB. The study included 103 culture proven, MDR-TB patients referred to the hospital during the study period (cases) and 103 randomly-selected TB patients with confirmed TB who turned negative after treatment (controls). Regressions analyses were used to determine the association of variables.

**Results:**

The mean age among cases and controls was 30.5 (±9.26) and 34.73 (±11.28) years, respectively. The likelihood of having MDR-TB was 20.3 times higher among those who had a any previous history of TB treatment (AOR=20.3 [CI 5.13, 80.58]), 15.7 times higher among those who had TB more than once (AOR=15.7 [CI 4.18, 58.71]) compared those who had once, 6.8 times higher among those who had pulmonary TB (AOR=6.8 [CI 1.16, 40.17]) and 16.1 times higher for those who had experienced treatment with a Category II regimen (AOR=16.1 [CI 2.40, 108.56]). HIV infection was less common among cases than controls.

**Conclusion:**

This study concluded that special attention should be given to patients with a history of the following: TB more than once, presence of pulmonary TB, and used a Category II treatment regimen, as these were all determining factors for MDR-TB. Thus, this study urges the development and implementation of well-planned and integrated strategies for MDR-TB control and prevention in Ethiopia.

## Introduction

The emergence of drug-resistant tuberculosis (TB) has become a serious public health threat in a number of countries [1]. The largest survey conducted by World Health Organization (WHO) in more than 80 countries in 2008 confirmed that the spread of MDR-TB had increased at an alarming rate, despite implementation of the directly observed therapy strategy (DOTS) since the 1990s [1–3]. More specifically, 5.3% of the world’s TB cases, or 510,000 people, were estimated to have multi drug-resistant TB (MDR-TB), which resulted in 110,000 deaths [4]. Generally, drug-resistant TB has become one of the major concerns of the antibiotic resistance pandemic [4]. The emergence of MDR-TB in Ethiopia is not a recent phenomenon. TB has been recognized as a major public health problem since the 1950s [5]. The WHO 2009 global TB report indicated that Ethiopia was one of the 27 high-burden MDR-TB countries ranked 15th with 5,979 estimated MDR-TB cases [5, 6]. An anti-TB drug resistance survey conducted in Ethiopia in 2007 showed the prevalence of MDR-TB was 1.6% among new cases and 11.8% among previously-treated TB cases [5].

The treatment of MDR-TB with second line drugs is long, complex process, and has a considerable rate of adverse effects [1, 5], making MDR-TB more costly and difficult to manage than drug-susceptible TB [1]. For example, the cost of drugs alone for treating the average MDR-TB patient is 50 to 200 times higher than for treating a drug-susceptible TB patient [7]. The cure rate for MDR TB is also reduced ranging from 6% to 59% [2].

Drug-resistant TB is considered to be a man-made problem resulting from the consequences of individual or combined factors related to the management of drug supply, patient management, and patient adherence. Behavioural and environmental factors, economic status, and poor infection control practices have also been identified as major contributing factors to the occurrence and spread of MDR-TB [3, 8, 9]. Nevertheless, the potential detrimental factors for the existing high rate of drug-resistant TB are not well known and the existing information is very often limited and scarce [7, 10]. As a result, the problem may continue to pose a serious public health threat for generations to come, unless local and contextual related solutions are devised. This study aims at producing reliable and valid information about the magnitude of the problem regarding factors contributing to the development of drug-resistant TB which may assist the Ministry of Health and potential stakeholders to improve the effectiveness of control interventions.

## Methods

**Study design and area:** a hospital-based, unmatched, case-control study design was carried out among cases (MDR-TB) and controls (TB) at St. Peter Hospital in Addis Ababa Ethiopia. St. Peter hospital is the largest TB referral center within the country. With several hundreds of patients welcomed for TB/HIV treatments, this facility has a long history of TB management. During the study period, St. Peter Hospital had about 363 MDR-TB patients and 121 TB patients being managed at inpatient and outpatient levels. St Peter Hospital has a strong collaborative partnership with the Ethiopian Health and Nutrition Research Institute (EHNRI), which hosts the TB National Reference Laboratory (NRL). Information obtained from the Health and Health Related Indicators revealed that a total of 35,002 TB and 6500 MDR-TB cases were registered within the country in the year 2013.

**Cases and controls:** cases (MDR- TB patients) were all culture-proven to be resistant to both isoniazid and rifampicin, with or without resistance to other anti-TB drugs whereas, controls (non-MDR TB patients) were all TB patients who were either not resistant to anti-TB drugs or resistant to isoniazid or rifampicin. Both cases and controls were under treatment at St. Peter Hospital.

**Sample size determination and sampling:** the required sample size was determined using EPI INFO software based on the assumption of a 26% of HIV exposure among controls (non-MDR-TB cases) [11], 80% power, 2.5 least odds ratio to be detected [11], 1 to 1 case to control ratio and 95% confidence level. After a 10% non-response rate was included, a total of 206 representative study subjects (103 cases and 103 controls) were selected using a systematic random sampling technique from clinically diagnosed TB and MDR-TB patients who were registered and being treated at St. Peter Hospital. Strict criteria and a conservative level of screening were applied to avoid misclassification bias between cases and controls. Culture and drug sensitivity tests were used to confirm MDR-TB cases and tests for acid fast bacilli (AFB) or clinical and radiological evidence were used for the verification of drug-sensitive TB cases. Patients were aged greater than eighteen years old, confirmed and registered to be either MDR-TB positive or non-MDR-TB positive during their treatment at St. Peter Hospital.

**Data collection:** primary data were collected from cases and controls by face-to-face interview using pretested structured questionnaires, whereas secondary data were collected by reviewing patient medical charts/ registration logs for the corresponding study participants. MDR-TB trained and well-experienced nurses currently working in other health institutions conducted the subject interviews and reviewed the corresponding records. Information on level of income and other variables not recorded in the medical charts were obtained by means of interview. The questionnaire was translated into Amharic and back-translated into English to ensure phrasing accuracy and validity. Further information on history and laboratory test results were obtained by studying each patient’s medical records. During data collection, data collectors used the N-95 respiratory mask (covers both the mouth and nose and filters more than 95% of particles). Study subjects wore a surgical mask to reduce the risk of TB transmission.

**Data management and analysis:** data were cleaned and entered using Epi Info version 3.5.1 and analyzed using SPSS version 20. Both descriptive and analytical statistical procedures were employed. Bivariate and multivariate logistic regression odds ratios, with a 95% confidence interval, were used to ascertain the association between the exposure variables and the outcome variable at the conventional 0.05 p-value level. Only exposure variables that were statistically significant variables at the bivariate level were entered into a multivariate logistic regression analysis to examine the main predictors of MDR-TB among the exposure variables. Non-significant variables were subsequently omitted in the modeling process. Once a satisfactory multivariate model was obtained, tests for interaction and confounding factors were conducted based on the likely combinations of the exposure variables. In addition, Pearson´s chi-square tests and odds ratio (OR) were used to assess the relationship between the exposure variables and MDR-TB.

**Ethical clearance:** ethical clearance was obtained from the research ethics committee of Gambi University College. Written consent was also obtained from St. Peter Hospital research center. Verbal consent was asked from each study subject. Subjects were also informed about the purpose of the study and respectfully asked to give genuine information. Confidentiality of the collected data was also ensured. Study subjects were also advised strictly not to terminate their TB treatment and follow-up under any circumstances.

## Results

Socio-demographic characteristics of study subjects: a total of 206 subjects participated in the study: 103 were cases and 103 were controls. Female study subjects constituted 52% of the cases and 38% of the controls. The mean age of respondents was 30.5 (+9.26) years among the case and 34.7 (+11.28) years among the control groups. With regard to self-reported monthly income, 42(40.8%) cases and 69 (67.0%) controls were earning less than or equal to $25. In terms of occupation, 89 (86.4%) cases and 63 (61.2%) controls were unemployed. The majority of the study subjects 141 (68.5%) were residing in Addis Ababa with their families 153 (74.3%) of cases stayed in a rented house compared to 104 (50.48%) controls. Bivariate analysis of socio- demographic characteristics showed that gender, average monthly income, occupational status, and residing place were significantly associated with MDR TB (Table 1).

**Table 1:** Socio-demographic characteristics and related factors for MDR-TB

Characteristics	Options	MDR-TB N=103	Non-MDR-TB N=103	Crude Odds ratio (COR), 95% CI	p- value
Gender	Male	49 (47.6%)	64 (62.1%)	1	**0.036**
	Female	54 (52.4%)	39 (37.9%)	1.8 (1.04, 3.15)	
Age (years)	≤24	16 (15.5%)	28 (27.2%)	1	0.15
	25-34	40 (38.8%)	41 (39.8%)	1.7 (0.80, 3.62)	
	35-44	29 (28.2%)	25 (24.3%)	2.0 (0.89, 4.58)	
	≥45	18 (17.5%)	9 (8.7%)	3.5 (1.27, 9.59)	
	Mean (±SD)	30.5 (±9.2)	34.7 (±11.2)		
Marital status	Single	47 (45.6%)	54 (52.4%)	1
	Married & living together	39 (37.9%)	31 (30.1%)	1.4 (0.78, 2.66)	0.23
	Married & living separately	9 (8.7%)	13 (12.6%)	7.9 (0.31, 2.02)
	Divorced	8 (7.8%)	5 (4.9%)	1.8 (0.58, 6.00)	
Religion	Orthodox	63 (61.2%)	67 (65.0%)	1
	Muslim	24 (23.3%)	24 (23.3%)	0.9 (0.48, 1.82)	0.855
	Protestant	14 (13.6%)	11 (10.7%)	0.7 (0.31, 1.75)	
	Catholic	2 (1.9%)	1 (1.0%)	0.4 (0.04, 5.31)	
Educational status	Unable to read & write	11(10.7%)	16 (15.5%)	0.5 (0.21, 1.51)	0.25
	Able to read & write	19 (18.4%)	3 (2.9%)	5.2 (1.34, 20.40)	
	Primary: Grades 1-6	14 (13.6%)	14 (13.6%)	0.8 (0.31, 2.15)	
	Grades 7-12	36 (35.0%)	51 (49.5%)	0.5 (0.27, 1.25)	
	> Grade 12	23 (22.3%)	19 (18.4%)	1	
Occupational status	Employed	14 (13.6%)	40 (38.8%)	1	
	Unemployed	89 (86.4%)	63 (61.2%)	4.0 (2.03, 8.04)	**0.001**
Income (per month)	≤$25	42 (40.8%)	69 (67.0%)	0.4 (0.18, 0.98)	
	$26-50	28 (27.2%)	14 (13.6%)	1.3 (0.51, 3.74)	
	$51 -100	17 (16.5%)	9 (8.7%)	1.3 (0.43, 3.96)	**0.047**
	>$100	16 (15.5%)	11 (10.7%)	1	
Residing in Addis Ababa	Yes	80 (77.7%)	61 (59.2%)	2.3 (1.30, 4.40)	**0.005**
	No	23 (22.3%)	42 (40.8%)	1	
Lived with	Family	74 (73.3%)	79 (77.5%)	1	
	Alone	25 (24.8%)	22 (21.6%)	1.2 (0.63, 2.33)	
	Other	2 (2.0%)	1 (1.0%)	2.1 (0.19, 24-04)	0.56
Living house	Owned	45 (43.7%)	42 (40.8%)	1	
	Rented	48 (46.6%)	56 (54.4%)	0.8 (0.45,1.41)	0.56
	Other	10 (9.7%)	5 (4.9%)	1.8 (0.58,5.91)	
Cooking room separated	Yes	53 (51.5%)	53 (51.5%)	1.0 (0.58,1.73)	0.9
	No	50 (48.5%)	50 (48.5%)	1	

Potential determinant factors for MDR-TB: the percentage of subjects with a history of TB treatment and pulmonary TB was greater for the cases than the controls. The percentage of subjects treated through the DOTS TB control strategy, imprisonment and with co-morbidity illness was similar between the groups. Results of bivariate analysis showed that the odds of developing MDR-TB were high among those who had previous history of TB, more than one episodes of TB, pulmonary TB, interrupted TB therapy, and been treated in category II. HIV co-infection and COPD were not associated with development of MDR-TB (Table 2).

**Table 2:** Potential determinant factors for MDR-TB at St. Peter hospital in Addis Ababa, Ethiopia

Clinical Characteristics	Options	MDR-TB N=103	Non- MDR-TB N=103	Bivariate Analysis COR, 95% CI	p-value
History of TB	Yes	96 (93.2%)	25 (24.3%)	42.79 (17.57,104.17)	**<0.001**
	No	7 (6.8%)	78 (75.7%)	1	
COPD	Yes	17 (17.7%)	21 (20.6%)	0.83 (0.41,1.69)	0.607
	No	79 (82%)	81 (79.4%)	1	
HIV infected	Yes	19 (18.4%)	48 (46.6%)	0.26 (0.14,0.49)	**<0.001**
	No	84 (81.6%)	55 (53.4%)	1	
TB episodes	One	34 (33.0%)	90 (87.4%)	1	
	Two or more	69 (67.0%)	13 (12.6%)	14.05 (6.89, 28.63)	**<0.001**
Biological site of TB	Pulmonary	92 (93.9%)	21 (80.8%)	3.65 (1.017,13.10)	**0.047**
	Extra-pulmonary	6 (6.1%)	5 (19.2%)	1	
Followed DOTs strategy	Yes	83 (80.6%)	92 (89.3%)	1	
	No	20 (19.4%)	11 (10.7%)	2.07 (0.486, 10.60)	0.08
Treatment category	Category I	48 (46.6%)	96 (94.1%)	1	
	Category II	39 (37.9%)	2 (2.0%)	39.0 (9.03, 168.37)	**0.045**
	Category III	5 (4.9%)	3 (2.9%)	3.33 (0.76, 14.54)	
	Category IV	11 (10.7%)	1 (1.0%)	2.82 (1.27, 175.44)	
Ever interrupted anti-TB drug therapy	Yes	24 (23.3%)	10 (9.7%)	2.82 (1.27, 6.26)	**0.011**
	No	79 (76.7%)	93 (90.3%)	1	
History of travel outside the country	Yes	88 (85.4%	90 (87.4%)	1.18 (0.53, 2.62)	0.68
	No	15 (14.6%)	13 (12.6%)	1	
History of contact with confirmed TB case	Yes	36 (35.6%)	31 (30.1%)	1	
	No	65 (64.4%)	72 (69.9%)	0.78 (0.43, 1.40)	0.40
Smoked cigarettes	Yes	16 (15.5%)	20 (19.4%)	0.76 (0.37, 1.57)	0.46
	No	87 (84.5%)	83 (80.6%)	1	
Consumed alcohol	Yes	30 (29.1%)	42 (40.8%)	1.29 (0.71, 2.31)	**0.08**
	No	73 (70.9%)	61 (59.2%)	1	
Chat chewed	Yes	24 (23.3%)	18 (17.5%)	1.43 (0.72, 2.84)	0.301
	No	79 (76.7%)	85 (82.5%)	1	

Multivariate logistic analysis of determinant factors of MDR-TB: results of multiple logistic regression analysis showed that a history of more than one episode of TB, pulmonary TB, and treatment category remained significantly associated with MDR-TB. The odds of MDR-TB were higher among those who had a history of previous TB treatment and multiple episodes of TB. The odds of MDR-TB was 6.83 times higher among those who had pulmonary TB than those with extra pulmonary TB (AOR=6.83 [CI 1.16, 40.17]). However, HIV co-infection was not associated with development of MDR-TB. The observed association between occupational status, residence, and income with MDR-TB in the bivariate analysis was not evident in the logistic regression analysis when controlled for all other variables in the model (Table 3).

**Table 3:** Multivariate logistic regression analysis of determinant factors for MDR-TB

Factors	Options	MDR TB N=103	Non MDR TB N=103	COR (95%CI)	Adjusted Odds Ratio (AOR) (95% CI)
Sex	Male	49(47.6%)	64(62.1%)	1	1
	Female	54(52.4%)	39(37.9%)	1.808(1.04,3.15)	3.05(0.94,9.84)
Had history of pervious TB	Yes	96(93.2%)	25(24.3%)	42.79(17.57,10.17)	20.35 (5.13, 80.58)+
	No	7(6.8%)	78(75.7%)	1	1
Occupational status	Employed	14(13.6%)	40(38.8%)	1	1
	Unemployed	89(86.4%)	63(61.2%)	4.04(2.03,8.04)	0.47(0.12,1.92)
Income	≤25$	42(40.8%)	69(67.0%)	0.42(0.18,0.99)	1.2(0.28,5.22)
	25-50$	28(27.2%)	14(13.6%)	1.38(0.51,3.74)	0.56(0.09,3.36)
	50-100$	17(16.5%)	9(8.7%)	1.30(0.43,3.96)	1.37(0.20,9.19)
	≥100$	16(15.5%)	11(10.7%)	1	1
Resided in Addis Ababa	Yes	80(77.7%)	61(59.2%)	1	1
	No	23(22.3%)	42(40.8%)	2.39(1.30,4.40)	2.04(0.65,6.38)
HIV infected	Yes	19(18.4%)	48(46.6%)	0.26 (0.13, 0.48)	0.65 (0.01, 0.28)+
	No	84(81.6%)	55(53.4%)	1	1
Episodes of TB	One	34(33.0%)	90(87.4%)	1	1
	More than1	9(67.0%)	13(12.6%)	14.06(6.89,28.63)	15.67 (4.18, 58.71)+	15.67 (4.18, 58.71)+
Biological TB site	Pulmonary	92(93.9%)	21(80.8%)	3.651(1.017,13.10)	6.83 (1.16, 40.17)+
	Extra pulmonary	6(6.1%)	5(19.2%)	1	1
Category of treatment	Category I	48(46.6%)	96(94.1%)	1	1
	Category II	39(37.9%)	2(2.0%)	39.0(9.03,168.37)	16.1 (2.40, 108.56)+
	Category III	5(4.9%)	3(2.9%)	3.3 (0.76,14.54)	2.4 (0.29,21.38)
	Category IV	11(10.7%)	1(1.0%)	22.0 (2.76,17.4)	7.7 (0.55,109.3)
Ever interrupted anti-TB drug	Yes	24(23.3)	10(9.7%)	2.8 (1.27,6.26)	0.2 (0.058,1.11)
	No	79(76.7%)	93(90.3%)	1	1

+ Indicates variable that showed significant association at p<0.05

## Discussion

The emergence of drug-resistant TB, particularly MDR-TB, is threatening to destabilize global TB control in a number of countries, including Ethiopia [12]. Either transmission of MDR TB strains or selection of single drug resistant strains may have contributed to the increased prevalence of MDR-TB [13]. Universal studies identified various socio-demographic, clinical, and behavioural risk factors as the main risk factors for drug-resistant TB [6, 14]. The Federal Ministry of Health in Ethiopia [6] also found that exposure to a known case of MDR-TB, a history of using poor quality TB drugs and treatment in a poorly performing control program had a positive association with MDR-TB. Similarly, in this study we also identified that history of previous treatment of TB, multiple episodes of TB, first site of TB infection being pulmonary, and Category II type of treatment were among the main determinant factors for the development of MDR-TB.

Previous treatment and episodes of TB: previous treatment has been widely recognized as the strongest risk factor for the development of MDR-TB [15, 16]. Usually the magnitude of drug-resistant isolates was higher among patients previously treated than among treatment-naive patients [1, 9]. In addition, if the treatment is unsuccessful, the prevalence is estimated to be up to 10 times higher [16]. Of course, this does not mean that treatment-naïve patients are not at risk of developing MDR-TB since the risk of transmission of resistant strains from close contacts is increasing day-by-day [17, 18].

Consistent with the results of previous studies both in developed and developing countries including Ethiopia [4, 14, 19, 20], this study showed that a history of prior TB treatment was a significant predictor of MDR-TB. The high association of previous TB treatment to MDR-TB may be attributed to inappropriate treatment regimens, inadequate or irregular drug supply, erratic and infrequent consumption of anti-TB drugs, unsatisfactory patient or clinician compliance, lack of supervision of treatment, and absence of infection control measures in hospitals [21]. The use of DOTS is highly recommended to promote treatment adherence [5, 22] but completing treatment for MDR-TB may be more challenging than completing first-line TB therapy, especially in resource-poor settings [23]. Recent studies also suggest that drug resistance is less likely to develop or be transmitted when TB patients are under DOTS [10, 23]. In the meantime, this study strongly suggests the need for exploring determinant factors for the inadequate treatment of TB patients all over the country.

Multiple episodes of TB: this study showed that having more than one TB episode significantly increased the risk of MDR-TB development. A similar study in Uganda also showed that multiple TB episodes and treatment failures were significantly associated with MDR-TB [24]. Previous treatment outcome, default, treatment failure or relapse may contribute to the increased risk. The possibility of patients to acquire MDR-TB initially would not also be undermined since exposure to known MDR-TB patients in closed or crowded places made them to acquire MDR-TB. There are suggestions that resistant infection may be transmitted directly from one individual to another [18] although there is limited published data on the probability of two household members with MDR-TB who share a similar genotype being more susceptible [10].

Biological site of TB: this study also found that smear-positive pulmonary TB patients had a higher risk of developing MDR-TB. The odd of MDR-TB was found to be 6.83 times higher among those who have pulmonary TB than extra pulmonary TB. This may also be associated with the fact that patients with smear-positive pulmonary TB have a higher bacterial load and may not respond to treatment within a short period of time compared with patients who have a low bacterial load [25]. It may also be associated with the limited capacities of the existing laboratory facilities and the difficulties of diagnosing the lower bacterial load of extra pulmonary MDR-TB.

Category II type of treatment: category II type of treatment is another significant variable that gives room for discussion. Multiple observational studies have examined outcomes among individuals receiving category II treatment and shown mixed results. Overall, success rates are in the 60% to 80% range [25, 26] with notably worse outcomes seen among patients who failed or relapsed after their initial treatment course [26]. The findings of this study however showed that individuals who were treated with the Category II regimen had an increased risk of MDR-TB. More than one explanation may be given for this observed association. Largely, management of patients who have been previously treated for TB has been a source of debate. In 1991, the WHO recommended use of the “category II retreatment regimen” for all patients with a prior history of TB treatment. The category II regimen added streptomycin to first-line agents and extended treatment to eight months. This addition in the regimen could change susceptible strains and lead to MDR-TB [27]. Besides, patients who fall into this category may have already developed MDR-TB at the time of initiation of the category II regimen.

HIV and AIDS: the role of HIV infection as a risk factor for the development of drug-resistant TB is also not clear [27]. Available information on this issue is limited and has been controversial for several years. High prevalence of drug resistance has been found among HIV patients [15]. However, a positive association between HIV and MDR-TB had not been reported from studies in East Africa including Ethiopia [20, 28]. In South Africa [29] and France [30], drug resistance was not associated with HIV infection. Similarly, the WHO Global Report on Resistance Surveillance did not find HIV to be a risk factor for MDR-TB [31]. The present study also did not find a significant association between HIV/AIDS co-infection and MDR-TB. However, lack of such an association may be caused by inadequate testing [31], therefore, more information about the characteristics of HIV-infected patients, including of CD4 count, viral load and antiretroviral therapy, is needed to understand this result. Smoking and alcohol consumption: although this study provided insufficient data to link socio-behavioural problems of TB patients like alcoholism and smoking as possible independent determinant factors for the development of MDR-TB, several studies have demonstrated they are important predictors of MDR-TB. For example, alcoholism was reported from Spain as an important predictor of MDR-TB [32]. Alcohol abuse and alcohol use disorders are known to play a role in the development of TB as well as in the outcomes of TB treatment [32]. However, the link between alcohol and MDR-TB may not be a direct causal relationship; instead, MDR-TB may be the result of interruptions in treatment, which are themselves attributable to the socio-behavioral problems of TB patients who regularly abuse alcohol [30, 33]. Patients who smoke are also likely to make other poor decisions with regard to their health, including being non-adherent to TB treatment [31]. Nevertheless, differences in methodology and place of study may be accountable for the existing discrepancy between our findings and others.

Socio-demographic characteristics: the authors did not find any significant association between socio-demographic variables (age, gender, educational status, religion, ethnicity, and others) and development of MDR-TB. However, the importance of socioeconomic determinants and other potential risk factors in the development of TB has been reiterated in numerous publications [32, 34]. More specifically, age is accepted by WHO as a risk factor for drug resistance [34] though the association of age and MDR-TB has not been consistently identified [23]. There have been previous study findings that support [16] and contradict [35] the association between MDR-TB and age. Similarly, difference in sex was reported to be a risk factor for MDR-TB in some studies [33] but not in several others [13, 20]. Studies regarding educational status also suggested that patients with little or no education reportedly were more likely to default or be noncompliant with anti-TB treatment, which might have led to the development of MDR-TB [36]. Ethnicity, on the other hand, was associated with an increased risk of developing MDR-TB regardless of living environment [37]. In addition, TB patients with a history of imprisonment were found to have a significantly increased risk of MDR-TB [37]. However, in this study we could not establish a link between history of imprisonment and development of MDR-TB.

Generally, in countries like Ethiopia, MDR-TB is becoming a challenge because of poor adherence to treatment and because patients stay in their communities for long periods of time without being diagnosed or seeking proper treatment. Even after diagnosis, there are few diagnostic and treatment facilities and lack of trained health professionals, and second line drugs; therefore, patients do not start treatment immediately. MDR-TB patients in rural and remote areas may not have access to health care services, may prefer consultation with traditional healers that are more readily available, and come late to health care centers. This delay potentially allows easy spread of the disease to a large number of individuals within a short time. Delayed recognition of drug resistance is one of the major factors contributing to MDR-TB [14].

Limitations: all data on education, living and employment conditions, history of imprisonment, use of alcohol, and smoking history were reported by patients participating in the study and were not verified. The possibility of minor reporting errors and the potential of recall bias cannot be ruled out.

## Conclusion

This study showed that history of previous TB, more than one TB episode, pulmonary type of TB, and treatment with a Category II regimen were significantly associated with MDR-TB. Therefore, health professionals involved with TB management should give priority to culture and DST for patients who have any of these risk factors to control the spread of drug-resistant TB in Ethiopia. The Ministry has to include TB guideline. Existing TB control measures need to address such risk factors to significantly reduce the development of MDR-TB. More meticulous administration of anti-TB medication as well as more efficient follow-up are required to prevent development of MDR-TB in the country. Lack of a positive association between MDR-TB and HIV co-infection observed in this study does not mean that the collaboration between TB and HIV control programs to provide greater support to co-infected patients not be strengthen. Rather, countrywide studies should be done to further explore risk factors for the development of MDR-TB.

### What is known about this topic

- Ethiopia is one of the high TB burden country and prone to MDR TB prevalence;
- MDR-TB is becoming a challenge because of poor adherence to treatment and because patients stay in their communities for long periods of time without being diagnosed or seeking proper treatment.

### What this study adds

- Lack of a positive association between MDR-TB and HIV co-infection;
- History of previous TB, more than one TB episode, pulmonary type of TB, and treatment with a Category II regimen the most significantly associated with MDR-TB.

## References

[1] WHO (2007). The Global MDR-TB and XDR-TB Response Plan 2007-2008..

[2] Sharma SK, Mohan A (2004). Multidrug-resistant tuberculosis. Indian J Med Res..

[3] (2008). Federal Ministry of Health of Ethiopia (FMOH): Tuberculosis, leprosy and TB/HIV prevention and control program (manual)..

[4] WHO (2009). WHO country Cooperation Strategy..

[5] WHO (2010). Drug Resistance Tuberculosis Now at Record Levels..

[6] (2009). Federal Ministry of Health (FMOH): Guideline for Program and Clinical Management of Drug Resistant Tuberculosis..

[7] Espinal MA, Kim SJ, Suarez PG, Kam KM, Khomenko AG, Migliori GB (2000). Standard short-course chemotherapy for drug-resistant tuberculosis: Treatment Outcomes in 6 Countries. Journal of the American Medical Association..

[8] Ireneau S, Fitzpatrick C, Dennis F, Bashir S, Peter A (2013). Multidrug resistant tuberculosis Somalia. Emerging Infectious Diseases..

[9] Skrahina A, Hurevich H, Zalutskaya A, Sahalchyk E, Astrauko A, Hoffner S (2010). Multidrug-resistant tuberculosis in Belarus: the size of the problem and associated risk factors. Bulletin of the World Health Organization..

[10] WHO (2006). Guidelines for the Programmatic Management of Drug-Resistant Tuberculosis..

[11] Tadesse F (2015). Risk factors for multidrug-resistant tuberculosis in Addis Ababa Ethiopia. Universal Journal of Public Health..

[12] Burki T (2010). Tuberculosis-resistance, funding, and drugs. Lancet Infect Dis..

[13] Yang X, Li Y, Wen X, Wu X, Li X (2010). Risk factors for drug resistance in pulmonary tuberculosis in patients. J Evid Based Med..

[14] Hirpa S, Medhin G, Girma B, Melese M, Mekonen A (2013). Determinants of multidrug-resistant tuberculosis in patients who underwent first-line treatment in Addis Ababa: a case control study. BMC Public Health..

[15] (2014). European Centre for Disease Prevention and Control: Rapid Risk Assessment: Healthcare system factors influencing treatment results of MDR TB patient..

[16] Suárez García I, Rodríguez Blanco A, Vidal Pérez JL, García Viejo MA, Jaras Hernández MJ (2009). Risk factors for multidrug-resistant tuberculosis in a tuberculosis unit in Madrid, Spain. Eur J Clin Microbiol Infect Dis..

[17] Flora MS, Amin MN, Karim MR, Afros S, Islam S, Alam A (2013). Risk factors of multi drug resistant tuberculosis in bangladeshi population: a case control study. Bangladesh Medical Research Council Bulletin..

[18] Duarte R, Santos A, Mota M, Carvalho A, Marques A, Barros H (2011). Involving community partners in the management of tuberculosis among drug users. Public Health..

[19] Abate D, Taye B, Abisino M, Bidglign S (2012). Epidemiology of anti-Tuberculosis drug resistance patterns and trends in tuberculosis referral hospital in Addis Ababa. Ethiopia BMC Res notes..

[20] Berhan A, Berhan Y, Yizengaw D (2013). A meta -analysis of drug resistant tuberculosis in Sub-Saharan Africa: how strongly associated with previous treatment and HIV co-infection?. Ethiop J Health Sci..

[21] Grant A, Gothard P, Thwaites G (2008). Managing drug resistant tuberculosis. BMJ..

[22] Moonan P, Quituga T, Pogoda J, Woo G, Drewyer G, Sahbazian B, Denise Dunbar, Kenneth Jost C, Charles Wallace, Stephen Weis E (2011). Does Directly Observed Therapy (DOT) Reduce Drug Resistant Tuberculosis?. BMC Public Health..

[23] Cohen T, Murray M, Abubakar I (2011). Multiple introductions of multidrug-resistant tuberculosis into households, Lima, Peru. Emerging Infectious Diseases..

[24] Kanaya AM, Glidden DV, Chambers HF (2001). Identifying pulmonary tuberculosis in patients with negative sputum smear results. Chest..

[25] WHO (2010). Treatment of Tuberculosis: Guidelines for National Programmes..

[26] Ormerod LP (2005). Multidrug-resistant tuberculosis (MDR-TB): epidemiology, prevention and treatment. Br Med Bull..

[27] Fischl MA, Uttamchandani RB, Daikos GL (1992). An outbreak of tuberculosis caused by multiple-drug-resistant tubercle bacilli among patients with HIV infection. Ann Intern Med..

[28] Akksilp S (2009). Multidrug-resistant TB and HIV in Thailand: overlapping, but not independently associated, risk factors. Southeast Asian J Trop Med Public Health..

[29] Weyer K, Brand J, Lancaster J, levin J, van der Walt M (2007). Determinants of multidrug-resistant tuberculosis in South Africa: results from a national survey 2007. S Afr Med J..

[30] WHO (2010). Multidrug and Extensively Drug-Resistant TB (M/XDR-TB)..

[31] Gomes M, Correia A, Mendonça D, Duarte R (2014). Risk factors for drug resistant tuberculosis. Journal of Tuberculosis Research..

[32] Rehm J, Samokhvalov AV, Neuman MG, Room R, Parry C, K L, Patra J, Poznyak V, Popova S (2009). The association between alcohol use, alcohol use disorders and tuberculosis (TB): a systematic review. BMC Public Health..

[33] Kidenya BR, Webster LE, Behan S, Kabangila R, Peck RN, Mshana SE, Ocheretina O, Fitzgerald DW (2013). Epidemiology and genetic diversity of multidrug-resistant tuberculosis in East Africa. Tuberculosis (Edinb)..

[34] Arinaminpathy N, Dye C (2010). Health in financial crises: economic recession and tuberculosis in Central and Eastern Europe. J R Soc Interface..

[35] Lee JH, Chang JH (2001). Drug-resistant tuberculosis in a tertiary referral teaching hospital of Korea. Korean J Intern Med..

[36] Dubrovina I, Miskinis K, Lyepshina S, Yann Y, Hoffmann H, Zaleskis R, Nunn P, Zignol M (2008). Drug-resistant tuberculosis and HIV in Ukraine: a threatening convergence of two epidemics?. Int J Tuberc Lung Dis..

[37] Kimerling ME, Slavuckij A, Chavers S, Peremtin GG, Tonkel T, Sirotkina O, Golubchikova V, Baddeley A (2003). The risk of MDR-TB and poly-resistant tuberculosis among the civilian population of Tomsk city, Siberia, 1999. Int J Tuberc Lung Dis..

